# Altered cortical bone strength and lean mass in young women with long-duration (19 years) type 1 diabetes

**DOI:** 10.1038/s41598-020-78853-7

**Published:** 2020-12-22

**Authors:** Daniel Novak, Gun Forsander, Eva Kristiansen, Anna Svedlund, Per Magnusson, Diana Swolin-Eide

**Affiliations:** 1 Department of Paediatrics, Institute for Clinical Sciences, Sahlgrenska Academy, University of Gothenburg, Gothenburg, Sweden; 2 Region Västra Götaland, Sahlgrenska University Hospital, Department of Paediatrics, Gothenburg, Sweden; 3 Department of Clinical Chemistry, and Department of Biomedical and Clinical Sciences, Linköping University, 581 85 Linköping, Sweden

**Keywords:** Diabetes, Diabetes complications, Type 1 diabetes, Bone, Bone imaging

## Abstract

To investigate bone health and body composition in young women with long-duration type 1 diabetes (T1D) in relation to matched controls. Twenty-three Swedish women, age 19.2–27.9 years, with a T1D duration of 10 years or more were recruited from the Swedish National Diabetes Registry (NDR). An age-, gender- and geography-matched control group was recruited. Bone mass and body composition were assessed by dual-energy X-ray absorptiometry and peripheral quantitative computed tomography. Data was retrieved from the NDR and SWEDIABKIDS registries. T1D individuals had a mean diabetes duration of 19 years. T1D individuals had reduced lean mass (40.0 ± 6.1 kg vs. 43.9 ± 4.9 kg) and were shorter (1.66 ± 0.06 m vs. 1.71 ± 0.06 m) although comparable BMI. Subjects with T1D had lower muscle area (*P* = 0.0045). No differences were observed for fractures; physical activity; total, lumbar spine or femur areal bone mineral density. The cortical bone strength strain index was lower for TD1 patients (1875 ± 399 mm^3^ vs. 2277 ± 332 mm^3^). In conclusion, young women with long-term diabetes duration showed reduced cortical bone strength, decreased periosteal circumference, endosteal circumference and altered body composition. These factors contribute to the health burden of TD1, which warrants further attention for advancing bone health in women with T1D.

## Introduction

An increase in the incidence of type 1 diabetes (T1D) has been observed worldwide in recent decades^1^. It is understood that T1D is a disease that involves environmental triggers acting with genetic susceptibility to initiate a destruction of pancreatic β-cells, in which an increasing role for environmental factors has been suggested for the disease aetiology^2^. Approximately 10% of all T1D cases are due to familial aggregation, but more than 20% when accounting for the extended family history^3^.

T1D is the second most common chronic disease amongst children and is associated with multiple secondary complications^4^. The risk of cardiovascular complications accelerates during adolescence, especially amongst women^5^. Female adolescents have a higher risk for diabetes ketoacidosis, dyslipidaemia and weight problems, and as a group, women smoke more than men^6,7^. Body composition of women with T1D compared with that of controls has shown a higher fat mass in the former^8^.

The treatment of children with T1D in Sweden has been regulated since 1982 by national guidelines, which thereafter have been regularly updated. The last version, from 2018, coincides with the guidelines of the International Society of Paediatric and Adolescent Diabetes (www.ispad.org). The original Swedish guidelines stated that the treatment should aim for normoglycaemia, with the ultimate goal of preserving a high quality of life and preventing acute and future vascular health complications. Diabetes is still, however, a complex disease. Besides social and psychological consequences, metabolic disturbances can affect multiple organs and body tissues^9^.

The importance of bone health is increasingly recognised; however, numerous aspects have to be considered, as bone mass is influenced by genetics, mechanical loading, physical activity, growth, vitamin D and hormones, as well as nutritional factors. Peak bone mass is achieved during early adulthood and serves as the “bone bank” for the remainder of life. The interpretation of bone mineral measurements is more complex in children than in adults since children are growing individuals^10^. Many diseases and treatments during childhood and adolescence affect the acquisition of bone mineral that contributes to optimal peak bone mass. The risk of fractures is increased in adult patients with both T1D and type 2 diabetes (T2D)^11,12^. The underlying pathophysiological mechanisms are complex^13^. Reports, comprising measurements by dual-energy X-ray absorptiometry (DXA) and peripheral quantitative computed tomography (pQCT), have demonstrated reduced bone mass in children with diabetes^14–16^. Disease duration is an important factor that contributes to negative effects on bone mass^13,17^. However, information is limited, especially regarding young women, on background factors influencing the elevated risk for reduced bone mass and the elevated risk for fractures.

Female patients with T1D have a higher risk for poor metabolic control and early micro- and macrovascular complications in comparison with age-matched men. Moreover, long disease duration contributes to the risk of low bone mass. We hypothesised that young women with long diabetes duration, but still without a history of fractures, would have lower bone mass and changed bone parameters compared with healthy controls.

The aim of the present study was to investigate bone health and body composition in young Swedish women patients with long-duration T1D of more than 10 years, with an age-, gender- and geography-matched healthy control group.

## Methods

### Subjects and study design

Caucasian women aged 19.2–27.9 years, with a long-term T1D duration of ≥ 10 years, who were former patients at The Queen Silvia Children’s Hospital in Gothenburg, Sweden, were recruited from the Swedish National Diabetes Registry (NDR). The NDR (www.ndr.nu) has been an integral part of diabetes care in Sweden since 1996 and is one of the largest, most comprehensive diabetes quality registers in the world. All patients had been transferred to an outpatient T1D diabetes clinic for adults, over the age of 18 years.

Exclusion criteria for participation in the study group were body mass index (BMI) ≥ 30 kg/m^2^, pregnancy, current breastfeeding, coeliac disease, thyroid disease and known metabolic bone disease. A total of 44 eligible women were contacted regarding their participation in this cross-sectional study.

An age-, gender- and geography-matched control group of healthy women, aged 19.2–27.6 years, were recruited randomly from hospital staff and university students. The study commenced in August 2017, and sampling was completed in September 2018.

### Measurements

#### Height and weight

Height was measured using a wall-mounted ruler to the nearest 0.1 cm (Ulmer stadiometer, Prof. Heinze, Ulm, Germany), and weight was measured on electronic scales to the nearest 0.1 kg with women dressed in light indoor clothing and without shoes (model 799; Seca GmbH & Co, KG, Hamburg, Germany) (Table 1).

**Table 1:** Clinical data of subjects with T1D and matched healthy controls.

	T1D (n = 23)	Controls (n = 23)	*P*-value
Age (years)	24.3 (2.7)	24.0 (2.6)	0.61
	24.7 (19.2; 27.9)	24.5 (19.2; 27.6)	
Weight (kg)	65.1 (9.8)	64.8 (6.3)	0.96
	67.7 (47.9; 86.3)	65.4 (53.8; 76.1)	
Height (m)	1.66 (0.06)	1.71 (0.06)	< 0.01
	1.66 (1.53; 1.81)	1.70 (1.62; 1.82)	
BMI (kg/m^2^)	23.7 (3.5)	22.3 (2.0)	0.13
	24.1 (17.4; 30.0)	22.2 (18.4; 26.3)	
Comorbidities (other than T1D)	4 (17.4%)	4 (17.4%)	1.00
Medications (other than insulin)	15 (65.2%)	5 (21.7%)	< 0.01
Supplements	7 (30.4%)	9 (39.1%)	0.76
Fracture	3 (13.0%) (digital, knee, wrist)	7 (30.4%) (digital, forearm, toe, knee, forearm, clavicular, forearm)	0.28
Smoking	2 (8.7%)	1 (4.3%)	1.00
Physical activity (IPAQ, MET-min/week)	3407 (2993)	3347 (3298)	0.68
	2739 (99; 9702)	2079 (392; 12,288)	
**Physical activity categories (high, moderate and low)**			0.67
High (> 3000 MET-min/week)	10 (43.5%)	8 (34.8%)	–
Moderate (600–3000 MET-min/week)	10 (43.5%)	12 (52.2%)	–
Low (< 600 MET-min/week)	3 (13.0%)	3 (13.0%)	–
Sedentary hours/day	6.26 (3.15)	7.28 (2.39)	0.24
	6.00 (1.50; 12.00)	8.00 (2.50; 11.00)	

For categorical variables n (%) is presented. For continuous variables, mean (SD)/median (minimum; maximum) are presented.

#### Registry data

Data regarding the T1D participants was retrieved from both the NDR and the national Swedish Paediatric Diabetes Quality Registries (SWEDIABKIDS). The SWEDIABKIDS register contains data on almost all children and adolescents (approximately 99%) under the age of 18 years who have diabetes in Sweden. Diabetes duration was calculated as the time from the first data entry at diagnosis into the SWEDIABKIDS registry to the study date. For each individual the most recent HbA1c value entered in the NDR and the average HbA1c of all the entries in the NDR since 18 years of age were used. We also calculated the mean (SD) and median (minimum; maximum) HbA1c levels for the three age intervals, 0–8.9, 9.0–13.9 and 14.0–18.0 years, to account for the glycaemic control during the years of growth. Retinopathy was grouped into four stages (Table 2). Data was retrieved from the registers regarding the most recent blood pressure reported from outpatient clinics to NDR, total cholesterol, low-density lipoprotein and microalbuminuria.

**Table 2:** Analyses of DXA and pQCT in subjects with T1D and matched healthy controls.

	T1D (n = 23)	Controls (n = 23)	Difference between groups, mean (95% CI)^a^	Unadjusted *P*-value	Adjusted *P*-value for physical activity and BMI	Adjusted *P*-value for physical activity, BMI and height
**DXA measurements**						
Total body BMC (g)	2424 (422)	2634 (263)	− 72 (− 223; 79)	0.049	0.025	0.34
	2372 (1772; 3231)	2642 (1910; 3121)				
Total body aBMD (g/cm^2^)	1.15 (0.14)	1.20 (0.09)	− 0.035 (− 0.101; 0.031)	0.19	0.046	0.29
	1.10 (0.94; 1.44)	1.19 (0.99; 1.36)				
Lumbar spine (L1-L4) BMC (g)	65.3 (14.2)	66.8 (9.2)	3.12 (− 3.45; 9.68)	0.68	0.66	0.34
	64.4 (45.3; 93.0)	66.8 (47.9; 83.5)				
Lumbar spine (L1-L4) aBMD (g/cm^2^)	1.28 (0.15)	1.26 (0.13)	0.043 (− 0.047; 0.134)	0.61	0.72	0.34
	1.28 (1.04; 1.58)	1.21 (1.03; 1.53)				
Total left femur aBMD (g/cm^2^)	1.05 (0.18)	1.11 (0.12)	− 0.038 (− 0.136; 0.060)	0.22	0.070	0.44
	1.02 (0.74; 1.53)	1.09 (0.84; 1.38)				
Mean total femur aBMD (g/cm^2^)	1.06 (0.18)	1.11 (0.12)	− 0.034 (− 0.128; 0.059)	0.26	0.090	0.46
	1.02 (0.76; 1.52)	1.08 (0.86; 1.37)				
Total fat mass (kg)	22.52 (5.88)	18.16 (3.64)	2.62 (0.52; 4.72)	0.0046	0.016	0.016
	21.70 (12.07; 36.14)	17.60 (11.80; 27.53)				
Total lean mass (kg)	39.96 (6.08)	43.93 (4.85)	− 2.50 (− 4.42; − 0.58)	0.018	0.0024	***0.012***
	40.40 (27.47; 50.75)	44.28 (35.88; 54.10)				
**pQCT measurements, tibia**						
Total area (mm^2^) (4%)	1048 (126)	1073 (124)	10.8 (− 75.5; 97.2)	0.50	0.56	0.80
	1046 (813; 1262)	1089 (766; 1303)				
Trabecular density (mg/cm^3^) (4%)	239.9 (30.6)	250.4 (39.8)	− 16.6 (− 40.1; 6.9)	0.32	0.12	0.16
	248.9 (168.3; 289.7)	245.0 (190.3; 343.3)				
Cortical density (mg/cm^3^) (66%)	1167 (19)	1152 (20)	16.4 (2.7; 30.1)	0.014	0.0029	0.020
	1160 (1132; 1198)	1157 (1109; 1192)				
Muscle density (mg/cm^3^) (66%)	78.3 (1.3)	77.1 (2.4)	1.09 (− 0.15; 2.33)	0.047	0.058	0.083
	78.4 (74.5; 80.9)	77.6 (70.4; 80.3)				
Muscle area (mm^2^) (66%)	5725 (912)	6535 (959)	− 720 (− 1203; − 237)	0.0053	0.0006	***0.0045***
	5996 (2728; 7143)	6166 (5358; 8922)				
Fat/muscle area ratio (%) (66%)	56.9 (17.3)	40.9 (9.0)	12.7 (2.9; 22.5)	0.0004	0.0018	0.012
	58.5 (25.2; 86.5)	42.1 (25.6; 58.4)				
Bone muscle area ratio (%) (66%)	6.10 (1.02)	5.77 (0.82)	0.522 (− 0.150; 1.194)	0.23	0.18	0.12
	6.00 (4.73; 9.29)	5.61 (4.67; 8.01)				
SSI (mm^3^) (66%)	1875 (399)	2277 (332)	− 305.5 (− 512.7; − 98.3)	0.0006	0.0007	***0.0049***
	1803 (1342; 2581)	2301 (1680; 2948)				
Cortical area (mm^2^) (66%)	276.8 (48.4)	305.5 (41.4)	− 35.0 (− 63.0; − 7.1)	0.036	0.015	0.18
	265.3 (216.8; 391.3)	297.0 (228.3; 384.8)				
Endosteal circumference (mm) (66%)	50.4 (4.7)	55.7 (5.0)	− 4.2 (− 7.7; − 0.8)	0.0006	0.0020	0.016
	50.9 (41.9; 60.0)	55.6 (46.3; 65.1)				
Periosteal circumference (mm) (66%)	77.5 (5.8)	83.3 (4.6)	− 4.2 (− 7.2; − 1.2)	0.0005	0.0007	***0.0070***
	76.5 (68.2; 89.7)	84.1 (74.9; 90.5)				
Cortical thickness (mm) (66%)	4.31 (0.55)	4.40 (0.56)	0.009 (− 0.322; 0.340)	0.60	0.29	0.96
	4.26 (3.56; 5.46)	4.32 (3.26; 5.33)				

For continuous variables, mean (SD)/median (minimum; maximum) are presented.

Bold and italic *P*-values remain significant after multiple comparison using the Hochberg step-up procedure for analyses adjusted for physical activity, BMI and height.

^a^Adjusted for physical activity, BMI and height.

### Questionnaires

A questionnaire was distributed to the participants regarding current diseases (except T1D), insulin treatment regimen and current medical treatment (except from insulin), use of supplements, previous fractures, and habits regarding the use of tobacco. The degree of physical activity during the previous week was assessed in both groups using a validated self-assessment form, i.e., the Swedish version of the International Physical Activity Questionnaire (IPAQ)^18^. Both questionnaires were completed in privacy during a clinical outpatient visit, and a research team member was present to answer any potential queries from the study participants.

### Assessment of bone mass and body composition

All measurements were performed at the Queen Silvia Children’s Hospital, Gothenburg, Sweden. Total body bone mineral density (BMD) and bone mineral content (BMC) were assessed by the Lunar iDXA (GE Lunar Corp., Madison, WI, USA). DXA measures bone in two dimensions, and this areal bone mineral density will be referred as aBMD (g/cm^2^) and not true volumetric bone mineral density. Calculated Z-scores are age- and gender-specific. Fat and lean body mass were also assessed by DXA.

The pQCT measurements were performed on the left tibia at 4% and 66% of the tibia length using the XCT 2000 (Stratec Medizintechnik GmbH, Pforzheim, Germany) with software version 6.00. A quality control calibration was performed before each measurement using a standard phantom and every 30 days using a cone phantom; both calibration tools were provided by the manufacturer. The tibial length was measured with a ruler from the medial malleolus to the medial tibial plateau. A voxel size of 0.5 mm and a scan speed of 20 mm/s were used. The exact position of the CT scans was defined in a coronal scout scan at the foot ankle joint. To reduce noise, the image was filtered using a median filter before the analysis. The performance of the device has been reported elsewhere^19^. All pQCT measurements in this study were re-evaluated by Stratec Medizintechnik GmbH. The polar strength strain index (SSI) of the cortex, which represents an estimation of the mechanical strength of the cortical bone in the measured tibia, was calculated by the software^20^.

### Statistical analysis

Power calculations were made regarding total body BMC. To detect a difference of 250 g (SD 272 g)^21^ with the power of 80% using the Mann–Whitney test for unpaired observations (significance level of 0.05); it was found that a sample size of 20 individuals was needed in each group. Descriptively, number and percentages were shown for categorical variables, and mean, SD, median, minimum and maximum were shown for continuous variables. Tests performed on both women with T1D and controls include Fisher’s exact test for dichotomous variables, Mantel–Haenszel Chi-square trend test for ordered categorical variables and Mann–Whitney U test for continuous variables. Unadjusted tests to determine the difference between the two groups with respect to DXA, pQCT and growth data were analysed using Student’s t-test. Analyses adjusted for physical activity and for BMI with and without height were performed using general linear models. Standard errors and test statistics were adjusted, and empirical sandwich estimators were applied in order to account for heteroscedasticity in data. The PROC GLIMMIX procedure in SAS software was used. Residual plots were reviewed and found to be satisfactory. Differences between the groups with 95% confidence intervals (CI) were presented. All analyses were performed using SAS software version 9.4 (SAS Institute Inc., Cary, NC, USA). All tests were two-tailed and conducted at the 0.05 significance level. We applied the multiple comparison step-up procedure of Hochberg^22^ that uses flexible testing sequence on secondary outcomes. The Hochberg step-up procedure organizes the individual *P*-values in order, from smallest to largest, and for the jth test in order, alpha(j) is calculated as alpha(T)/(k − j + 1), where alpha (T) is 0.05, k = total number of test and j is the current test in the order.

### Human rights and informed consent

The study was approved by the regional research ethics committee of the University of Gothenburg (no. 074-18) and conducted in accordance with the 1964 Helsinki declaration and its later amendments or comparable ethical standards. All participants received both written and oral information prior to study entry, and written informed consent was obtained from all subjects.

## Results

Forty-four young women with T1D fulfilling the inclusion criteria of being 19–28 years of age and with a T1D duration of more than 10 years were initially identified. Twenty-one of these patients were excluded because of existing exclusion criteria or due to practical problems. Finally, 23 women with T1D and 23 healthy matched controls were enrolled for completion of two questionnaires and assessments of body composition and bone mass by DXA and pQCT. Clinical data and characteristics of the study groups, i.e., individuals with T1D and healthy matched controls, are presented in Table 1.

### Body composition

Participants in both groups estimated that they performed the same degree of physical activity, T1D (mean ± SD) 3407 ± 2993 MET-min/week vs. controls 3347 ± 3298 MET-min/week, and had the same number of sedentary hours per day (Table 1). No significant differences were found between the study groups for age, weight SDS and BMI SDS. Women with T1D were significantly shorter than individuals in the control group (Table 1). Even though there was no statistical difference in BMI or BMI SDS between patients and controls, women with T1D had a lower total lean mass (39.96 ± 6.08 kg vs. 43.93 ± 4.85 kg, adjusted *P* = 0.012, even after multiple comparison by Hochberg). This finding coincides with the pQCT data showing that T1D patients had significantly less muscle area (*P* = 0.0045). A higher total fat mass was found in the T1D group (22.52 ± 5.88 kg vs 18.16 ± 3.64 kg, adjusted *P* = 0.016; however, this difference was not significant after Hochberg multiple comparison), measured by DXA (Table 2).

### Clinical data

A majority of the T1D subjects had a diabetes onset age below 10 years, and the average diabetes duration was 18.9 years (Table 3). The average HbA1c value during the last visit at the adult diabetes outpatient clinic was 68 mmol/mol (8.4%), and 15 patients (65%) showed signs of vascular complications. The mean levels of HbA1c increased gradually with increasing age. Five T1D individuals had blood pressure values defined as hypertension (i.e., ≥ 130/80 mmHg). More than half of the women with T1D had some degree of retinopathy. Apart from insulin, women with T1D used more drugs than the matched controls (*P* = 0.0067). Oral contraceptives were more commonly used within the T1D group.

**Table 3:** Clinical data and biochemical assessments of subjects with T1D.

	T1D (n = 23)
Age at diabetes onset (years)	5.7 (2.8)
	5.0 (1.0; 11.0)
Diabetes duration (years)	18.9 (2.2)
	18.5 (15.8; 23.0)
Insulin MDI	12 (52.2%)
Insulin pump	11 (47.8%)
Last HbA1c measurement (mmol/mol)	68.1 (13.0)
	63.0 (49.0; 94.0)
HbA1c since 18 years of age (mmol/mol)	72.1 (15.8)
	69.2 (49.1; 108.5)
HbA1c, 0–8.9 years (mmol/mol)	59.3 (12.3)
	58.8 (25.5; 106.9)
HbA1c, 9.0–13.9 year (mmol/mol)	64.3 (11.9)
	64.0 (31.3; 94.0)
HbA1c, 14.0–18.0 years (mmol/mol)	69.7 (13.8)
	69.2 (35.3; 107.0)
Microalbuminuria	0 (0.0%)
eGFR (MDRD)	111.1 (23.9)
	108.4 (71.7; 178.3) *n* = *22*
Retinopathy	15 (65.2%)
**Retinopathy stages**	
None	8 (34.8%)
Simplex	8 (34.8%)
Maculopathy	5 (21.7%)
Proliferative diabetes retinopathy	2 (8.7%)
Total cholesterol (mmol/L)	4.45 (0.64)
	4.45 (3.30; 5.70) *n* = 22
Low-density lipoprotein (mmol/L)	2.36 (0.52)
	2.36 (1.49; 3.85) *n* = 22
Systolic blood pressure (mmHg)	112.5 (10.0)
	110.0 (95.0; 136.0)
Diastolic blood pressure (mmHg)	68.0 (10.0)
	70.0 (40.0; 82.0)
High blood pressure (≥ 130/80 mmHg)	5 (21.7%)

For categorical variables, n (%) is presented. For continuous variables, mean (SD)/median (minimum; maximum) are presented.

### Bone mass parameters

Results for the bone mass parameters are presented in Table 2. No differences were found between the T1D and control groups for total body aBMD (mean ± SD) (1.15 ± 0.14 g/cm^2^ vs. 1.20 ± 0.09 g/cm^2^), lumbar spine aBMD (1.28 ± 0.15 g/cm^2^ vs. 1.26 ± 0.13 g/cm^2^) and mean total femur aBMD (1.06 ± 0.18 g/cm^2^ vs. 1.11 ± 0.12 g/cm^2^), or for lumbar spine BMC (65.3 ± 14.2 g vs. 66.8 ± 9.2 g), when adjusting for physical activity, BMI and height. Total body BMC was lower in the T1D group in comparison with the control group (2424 ± 422 g vs. 2634 ± 263 g); however, this difference was no longer apparent when total body BMC was adjusted for physical activity, BMI and height.

Results from the pQCT measurements did not reveal any differences between the T1D and control groups for trabecular density and cortical thickness. A higher cortical density (*P* = 0.020, adjusted for physical activity, BMI and height) was observed for the T1D group; however, this difference was not significant after Hochberg multiple comparison. The bone strength index of cortical bone, SSI, was lower amongst women with T1D (*P* = 0.0049, adjusted for physical activity, BMI and height, even after multiple comparison by Hochberg). In comparison with the control group, the cortical bone area was lower in the T1D group, albeit that this difference was not evident when data was adjusted for height. Reduced periosteal and endosteal circumferences were found for the T1D group. The discrepancy in periosteal circumference was apparent even after adjustment for physical activity, BMI and height (*P* = 0.0070), and after Hochberg multiple comparison (Table 2).

No correlation was observed between the duration of diabetes and total aBMD, pQCT trabecular density and pQCT cortical density. No difference was found between the T1D and control groups regarding fracture incidence, n = 3 (13.0%) and n = 7 (30.4%), respectively. Specific information about sustained fractures are presented in Table 1.

## Discussion

In general, young women with T1D have a less favourable metabolic control than men and have, as a group, a higher risk of future complications^5,23^. This study was designed to investigate bone health and body composition in young women with long-duration T1D (on average 19 years) in relation to well-matched controls. The main findings were that T1D individuals had changed body composition with reduced lean mass although comparable BMI. This study also demonstrated decreased bone strength index of cortical bone, i.e., SSI, and decreased periosteal circumference by pQCT. These findings contribute to the future health burden and warrant further attention in terms of efforts to improve bone health.

The recommended HbA1c value in Sweden for young individuals is currently $\le$ 48 mmol/mol (6.5%). The metabolic control was highly suboptimal in our study group, with a final average HbA1c value of 68 mmol/mol (8.4%). This could be compared with the current average HbA1c of 60 mmol/mol (7.6%) for all women in that age group with T1D in Sweden in 2018 (www.ndr.nu). Their suboptimal or poor metabolic control was reflected in the prevalence of vascular complications. Smoking is associated with lower BMD^24^ and is also associated with increased cardiovascular risk, which is why it is important to discuss smoking habits amongst young individuals with T1D.

In our study, women with T1D were shorter than controls, which could be explained either by suboptimal glycaemic control during the vulnerable adolescent growth spurt period or by chance because of unmatched participants regarding height in this relatively small study. Growth retardation is a well-known complication in patients with T1D^25^. Insulin deficiency may affect the quality of bone, especially if there is a lack of insulin during the intensive stages of bone growth. Unfortunately, the women with T1D in our study had suboptimal metabolic control during their intensive growth period (Table 3), which may have affected their final height. The HbA1c increased steadily with increasing age, which is a pattern still seen today (SWEDIABKIDS) and which portrays the difficulties in maintaining good metabolic control during a period with increased hormonal production and increased growth velocity. This is also a sensitive period during which the gain in bone mass is most rapid^26^.

In the study of a population-based cohort with data from 1994 to 2012, in which 30,394 individuals (aged 0–89 years) with T1D participated and were compared with a matched control group, it was concluded that T1D was associated with increased risk of fracture incidence and that individuals with T1D sustained a greater number of lower-extremity fractures^27^. Less is known about whether the degree of glycaemic control specifically plays a role in the fracture risk associated with diabetes. Blood glucose control appears to play a role in the known risk of fracture in diabetes; however, the effect is significant only in T1D cases, suggesting insulin deficiency and poor glycaemic control earlier in life could take a toll on bone mass amongst these patients^28^. A large, recent meta-analysis of genome-wide association studies found that decreased BMD has a profound effect on future fracture risk, which implies that investigating BMD and bone health in patients with diabetes would be of great interest^29^.

A paper by Liu et al.^30^ indicated that women with T1D show differences in BMD early in life, with significant differences present in females 20–37 years of age. It has recently been demonstrated that elevated blood glucose has a negative effect on bone before adulthood in patients with T1D, although no signs of osteoporosis were identified by DXA^31^. Lower hip BMD in these young women may explain, in part, the higher incidence of hip fracture experienced in postmenopausal women with T1D. In contrast, results from the current study do not indicate a difference in total aBMD, femur or lumbar spine aBMD for patients with T1D. Total BMC was reduced in T1D but not significantly different when adjusted for BMI, physical activity and height, which reflects the height difference in this group.

Reduced periosteal and endosteal circumferences were observed for the T1D group; and the smaller periosteal circumference was also evident after adjustments for physical activity, BMI, height, and Hochberg multiple comparison. In theory, smaller bone circumferences, despite similar cortical thickness and cortical area after adjustments for height, might in part explain why individuals with diabetes have an elevated risk for fractures. Bone mass that is further displaced from the central bending axis (i.e., wider bone width), results in an increased cross-sectional moment of inertia, which confers greater resistance to bending^32^. The presented results are in line with Saha et al.^33^ who recently showed cortical bone size deficits (assessed by pQCT) in postmenopausal women with T1D onset before the age of 20 years. Weber et al.^34^ investigated skeletal outcomes within the first year of T1D diagnosis in children (7 to 17 years) and found decreased tibia cortical volumetric BMD in addition to lower rates of bone accrual, which was associated with poor glycemic control.

A study by Saha et al.^35^ found that T1D is associated with decreased BMC which could affect cross-sectional size and cortical rigidity; the study also found that male adolescents were more prone to these changes. The estimate of bone strength index of cortical bone, SSI, was reduced in patients with T1D, which suggests that the skeletal microstructure is altered in an unfavourable way that could contribute to the increased fracture risk at a greater age. In a study by Nilsson et al.^36^ of elderly women with T2D, a more favourable bone microarchitecture was observed. No difference in adjusted aBMD was observed in the population compared with healthy controls. As in our study, Nilsson et al.^36^ found a reduced bone material strength index; they hypothesized that this, together with impaired physical function, may explain the increased fracture risk in women with diabetes. The pathophysiological mechanisms of diabetes-induced bone fragility are complex. On the molecular level, it has been demonstrated that accumulation of advanced glycation end products compromise collagen properties, which leads to abnormal biomechanical properties associated with reduced bone strength^13^.

Our study confirmed earlier findings with a changed body composition in individuals with T1D regarding lower lean mass. A Swedish study evaluating body composition by DXA of adolescent women with T1D compared with controls showed higher total fat mass amongst women with T1D than amongst the controls^8^. The increased fat mass was associated with a higher insulin requirement, poor metabolic control and increased blood lipids. In the present study, there was no difference in physical exercise or sedentary hours that could explain the difference in lean mass. In our study, however, the women with T1D had poor metabolic control, which is associated with higher insulin resistance and a higher insulin requirement; this may have affected their body composition.

The current study has a number of strengths, such as a well-matched control group, validated data from national paediatric and adult diabetes registers, and the long follow-up period, on average 19 years, from diabetes diagnosis. Furthermore, two- and three-dimensional bone measurement techniques by DXA and pQCT were used. However, the study also possesses some limitations that warrant consideration when interpreting the data, such as the relatively low number of patients. The use of accelerometers to monitor physical activity would have been preferable to the IPAQ questionnaire that was used, even though the IPAQ is a validated self-assessment form used frequently to assess the amount of physical activity.

In conclusion, young women with long-term T1D duration showed altered body composition and decreased cortical bone strength in comparison with controls. A changed body composition and affected bone parameters at this young age increase the health burden in T1D. Preventive interventions to improve metabolic control and bone health are of great importance for future healthcare, aiming at optimal health-related quality of life.

## Abbreviations

BMC: Bone mineral content
BMD: Bone mineral density
aBMD: Areal bone mineral density
BMI: Body mass index
DXA: Dual-energy X-ray absorptiometry
pQCT: Peripheral quantitative computed tomography
T1D: Type 1 diabetes
T2D: Type 2 diabetes
NDR: National Diabetes Registry
SWEDIABKIDS: The Swedish Paediatric National Diabetes Registry for Children 0–18 years
SSI: Strength strain index
